# Pretreatment Glasgow prognostic score predicts survival among patients with high PD‐L1 expression administered first‐line pembrolizumab monotherapy for non‐small cell lung cancer

**DOI:** 10.1002/cam4.4220

**Published:** 2021-08-20

**Authors:** Hisao Imai, Takayuki Kishikawa, Hiroyuki Minemura, Yutaka Yamada, Tatsuya Ibe, Ou Yamaguchi, Atsuto Mouri, Yoichiro Hamamoto, Kenya Kanazawa, Takashi Kasai, Kyoichi Kaira, Takayuki Kaburagi, Koichi Minato, Kunihiko Kobayashi, Hiroshi Kagamu

**Affiliations:** ^1^ Department of Respiratory Medicine Comprehensive Cancer Center International Medical Center Saitama Medical University Hidaka Saitama Japan; ^2^ Division of Respiratory Medicine Gunma Prefectural Cancer Center Ota Gunma Japan; ^3^ Division of Thoracic Oncology Tochigi Cancer Center Utsunomiya Tochigi Japan; ^4^ Department of Pulmonary Medicine Fukushima Medical University Fukushima Japan; ^5^ Division of Respiratory Medicine Ibaraki Prefectural Central Hospital Kasama Ibaraki Japan; ^6^ Department of Pulmonary Medicine National Hospital Organization Nishisaitama‐Chuo National Hospital Tokorozawa Saitama Japan

**Keywords:** body mass index, Glasgow prognostic score, non‐small cell lung cancer, pembrolizumab

## Abstract

**Background:**

There are no established biomarkers for predicting the efficacy of first‐line pembrolizumab monotherapy in patients with high programmed death‐ligand 1 (PD‐L1) expression. In this study, we investigated whether the Glasgow prognostic score (GPS), neutrophil‐to‐lymphocyte ratio (NLR), and body mass index (BMI) can be used to evaluate the effect of first‐line pembrolizumab monotherapy in patients with advanced non‐small cell lung cancer (NSCLC) who express high levels of PD‐L1.

**Methods:**

We reviewed data from 142 patients with high PD‐L1 expression who underwent first‐line pembrolizumab monotherapy for NSCLC at six Japanese institutions between February 2017 and June 2019 and assessed the prognostic value of the GPS, NLR, and BMI. The Kaplan–Meier method and Cox proportional hazard models were used to examine differences in progression‐free survival (PFS) and overall survival (OS). The GPS, NLR, and BMI were calculated using C‐reactive protein and albumin concentrations, neutrophil and lymphocyte counts, and body weight and height, respectively.

**Results:**

The GPS independently predicted the first‐line pembrolizumab monotherapy efficacy, as a good GPS (GPS 0–1) was associated with a significantly better PFS and OS compared to a poor GPS (GPS 2) (PFS: 11.8 vs. 2.9 months, *p* < 0.0001; OS: not reached vs. 8.3 months, *p* < 0.0001). Furthermore, BMI independently predicted efficacy, as patients with high BMI (BMI ≥21.4) exhibited significantly better OS compared to those with low BMI (BMI <21.4) (OS: not reached vs. 14.1 months, *p* = 0.006).

**Conclusions:**

Among patients with high PD‐L1 expression undergoing first‐line pembrolizumab monotherapy for NSCLC, the GPS is significantly correlated with both PFS and OS, and BMI with OS, indicating that they could be used to predict treatment outcome in these patients. To the best of our knowledge, this is the first study to assess the relationship among the GPS, NLR, and BMI and survival among patients with high PD‐L1 expression undergoing first‐line pembrolizumab monotherapy for NSCLC.

## Graphical abstract

We investigated whether the Glasgow prognostic score (GPS), neutrophil‐to‐lymphocyte ratio, and body mass index (BMI) can be used to evaluate the effect of first‐line pembrolizumab monotherapy in patients with advanced non‐small cell lung cancer (NSCLC) who express high levels of programmed death‐ligand 1 (PD‐L1). Among patients with high PD‐L1 expression undergoing first‐line pembrolizumab monotherapy for NSCLC, the GPS is significantly correlated with both progression‐free survival and overall survival (OS), and BMI with OS, indicating that they could be used to predict treatment outcome in these patients.

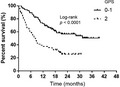

## 1 INTRODUCTION

Lung cancer is the leading cause of cancer‐related deaths globally, and non‐small cell lung cancer (NSCLC) accounts for approximately 85% of all lung cancers.^1^ A previous open‐label phase III trial revealed that pembrolizumab monotherapy is an effective first‐line treatment for patients with NSCLC with high programmed death‐ligand 1 (PD‐L1) expression (≥50% of tumor cells).^2^ Thus, pembrolizumab monotherapy is now considered a standard first‐line treatment for patients with high PD‐L1 expression and with no contraindications to immune checkpoint inhibitors (ICIs).

Most patients with NSCLC are diagnosed at an advanced stage, and these patients frequently experience weight loss and a systemic inflammatory response (SIR), which influences cancer cachexia.^3^, ^4^ Thus, cancer‐related prognosis is examined using various SIR‐based scoring systems, such as the Glasgow prognostic score (GPS) and neutrophil‐to‐lymphocyte ratio (NLR). The GPS is a SIR‐based scoring system that comprises serum C‐reactive protein (CRP) and albumin concentrations.^3^ The GPS is an independent prognostic marker for advanced NSCLC.^5^, ^6^, ^7^, ^8^, ^9^, ^10^, ^11^, ^12^, ^13^, ^14^ Although several studies have reported on the relationship between the GPS and ICI treatment efficacy in NSCLC for different lines of treatment, various ICIs, and various levels of PD‐L1 expression,^14^, ^15^ no studies have evaluated the relationship between the GPS and the efficacy of first‐line pembrolizumab monotherapy for NSCLC in patients with high PD‐L1 expression. SIR‐based markers can predict the response to ICIs, with NLR predicting the response to ICIs in melanoma,^16^, ^17^, ^18^ renal cell carcinoma,^19^ and NSCLC.^20^, ^21^, ^22^ Additionally, body mass index (BMI) has been reported as a prognostic marker for various malignancies. The presence of sarcopenia was negatively associated with outcomes in patients with NSCLC receiving ICI.^23^ Additionally, BMI is associated with ICI treatment outcomes in solid tumors, including melanoma, renal cell cancer, and NSCLC.^24^ However, there is limited data regarding the relationship between the GPS, NLR, and BMI and response to first‐line pembrolizumab monotherapy for NSCLC with high PD‐L1 expression. A recent study reported a relationship between BMI and the effect of ICIs in NSCLC.^25^ When a BMI cutoff value of 22 kg/m^2^ was used, no significant difference was observed in the progression‐free survival (PFS) or overall survival (OS) between high‐ and low‐BMI groups among patients with NSCLC with high PD‐L1 expression (≥50%) who were treated with pembrolizumab as a first‐line therapy. However, in patients with NSCLC treated with nivolumab/pembrolizumab/atezolizumab as a second‐ or later‐line treatment, survival was significantly longer in patients with a high BMI versus those with a low BMI. Thus, the relationship between BMI and the efficacy of ICIs in NSCLC is unclear. Therefore, in the current study, we assessed whether the GPS, NLR, and BMI could predict the response to first‐line pembrolizumab monotherapy in patients with NSCLC and high PD‐L1 expression.

## 2 METHODS

### 2.1 Patients

This retrospective study assessed the clinical effects of first‐line pembrolizumab monotherapy in 144 patients with NSCLC and high PD‐L1 expression at six Japanese institutions between February 2017 and June 2019. Among them, pretreatment albumin and CRP values were missing in two patients. Thus, 142 patients were included in the analysis. The NSCLC was histologically classified using the 2015 World Health Organization system and staged using version 8 of the Tumor–Node–Metastasis staging system. The eligibility criteria were as follows: (1) histologically or cytologically confirmed NSCLC, (2) unresectable stage III/IV disease or postoperative recurrence, and (3) high PD‐L1 expression (≥50% of tumor cells). The patients received first‐line treatment with pembrolizumab monotherapy (200 mg), and a confirmation of a censored event or death was made for each patient. Pretreatment Tumor–Node–Metastasis staging was based on physical examination, chest radiography, thoracic and abdominal computed tomography, brain computed tomography or magnetic resonance imaging, and bone scintigraphy or ^18^F‐fluorodeoxyglucose positron emission tomography. We reviewed the patient charts to collect data regarding baseline characteristics and response to first‐line pembrolizumab monotherapy. The study design was approved by the Institutional Review Board of each participating institution. The requirement for informed consent was waived owing to the retrospective nature of the study.

### 2.2 Assessment of PD‐L1 expression

PD‐L1 expression in formalin‐fixed tumor specimens was evaluated using a commercially available immunohistochemistry kit for detecting PD‐L1 (22C3 pharmDx assay; Dako North America).^26^ Biopsy specimens from the time of lung cancer diagnosis or from the time of initiation of pembrolizumab monotherapy were collected from the institutional archives. PD‐L1 expression (membranous staining) was quantified as the proportion of positive cells among the tumor cells and tumor‐infiltrating immune cells.

### 2.3 Treatment

The patients included in the study had not previously received ICI therapy; they received first‐line treatment with pembrolizumab monotherapy (200 mg intravenously once every 3 weeks), which was continued until disease progression, unacceptable toxicity, or withdrawal of consent.

### 2.4 Assessment of treatment efficacy

Serum CRP and albumin levels as well as neutrophil and lymphocyte counts were measured at treatment initiation. Blood samples were usually collected on the day before pembrolizumab administration or on the day of administration. The GPS values were defined as: a GPS of 0 (CRP <1.0 mg/dl and albumin >3.5 mg/dl), a GPS of 1 (CRP ≥1.0 mg/dl or albumin <3.5 mg/dl), or a GPS of 2 (CRP ≥1.0 mg/dl and albumin <3.5 mg/dl). NLR was defined as the ratio of absolute neutrophil and absolute lymphocyte counts; the NLR cut‐off value was set at 5.^20^, ^27^ BMI, which was determined at treatment initiation, was defined as the weight (kg) divided by the height (m) squared. The patients were stratified into BMI groups, as defined by the receiver operating characteristic (ROC) curve: low‐weight group (BMI <21.4 kg/m^2^) and high‐weight group (BMI ≥21.4 kg/m^2^). The optimal cut‐off value that differentiated high BMI from low BMI, as determined by the ROC curve analysis for PFS, was 21.4 (AUC: 0.578; sensitivity: 68.2%; specificity: 48.5%).

Tumor response was quantified as the best overall response and maximum tumor shrinkage. Radiological tumor responses were assessed according to the Response Evaluation Criteria in Solid Tumors (version 1.1): disappearance of all target lesions (complete response [CR]); a ≥30% decrease in the sum of the target lesion diameters relative to the baseline (partial response [PR]), a ≥20% increase in the sum of the target lesion diameters relative to the smallest value during the study period (progressive disease [PD]), and insufficient shrinkage for being qualified as PR and insufficient growth for being qualified as PD (stable disease [SD]).^28^ The PFS interval was calculated from the start of pembrolizumab monotherapy until the first instance of PD or death from any cause. The OS interval was calculated from the start of pembrolizumab monotherapy until the first instance of death or censoring at the last follow‐up.

### 2.5 Statistical analyses

Categorical and continuous variables were analyzed using Fisher's exact test and Welch's *t*‐test, respectively. A Cox proportional hazards model with stepwise regression was used to identify factors that predicted PFS and OS, and the results were described as hazard ratios (HRs) and 95% confidence intervals (CIs). PFS and OS were compared using the log‐rank test. Differences were considered statistically significant at a two‐tailed *p* ≤ 0.05. All analyses were conducted using the JMP software for Windows, version 11.0 (SAS Institute).

## 3 RESULTS

### 3.1 Patient characteristics and treatment efficacy

Table 1 presents the characteristics of the 142 patients who received pembrolizumab monotherapy; they included 117 men (82.4%) and 25 women (17.6%), with a median age of 70 years (range, 47–86 years). The Eastern Cooperative Oncology Group (ECOG)‐performance status (PS) scores were 0–1 for 110 patients (77.4%) and 2–3 for 32 patients (22.6%). Adenocarcinoma was observed in 75 of the 142 patients (52.8%). A total of 123 patients (86.6%) had stage III–IV disease. Nineteen patients (13.4%) experienced postoperative recurrence. All patients presented high PD‐L1 expression (≥50% of the tumor cells). The driver gene mutation/translocation status of the patients was wild type, negative, or unknown. The median number of pembrolizumab cycles was five (range, 1–55), and the responses to treatment among all patients were classified as CR (*n* = 1), PR (*n* = 60), SD (*n* = 44), and PD (*n* = 25). The overall response rate was 42.9% (95% CI: 34.8–51.0), and the disease control rate was 73.9% (95% CI: 66.7–81.1).

**TABLE 1:** Patient characteristics

Variables	All patients
Patients (*n*)	142
Characteristics	
Gender	
Male/female	117/25
Median age at treatment (years) [range]	70 (47–86)
PS	
0/1/2/3/4	48/62/23/9/0
Smoking history	
Yes/No	130/12
Histology	
Adenocarcinoma/Squamous cell carcinoma/others	75/40/27
Clinical stage at diagnosis	
III/IV/postoperative recurrence	18/105/19
PD‐L1 TPS (%)	
50–89/90–100	85/57
Driver mutation/translocation	
EGFR/ALK/WT, negative, unknown	0/0/142
Intracranial metastases at initial treatment	
Yes/No	34/108
Liver metastases at initial treatment	
Yes/No	11/131
Bone metastases at initial treatment	
Yes/No	44/98
BMI (kg/m^2^)	
Median (range)	20.3 (14.1–31.7)
Prior radiotherapy	
Yes/No	45/97
Administration cycles of pembrolizumab	
Median (range)	5 (1–55)
Tumor response	
Complete response	1
Partial response	60
Stable disease	44
Progressive disease	25
Not evaluated	12
Response rate (%) (95% CI)	42.9 (34.8–51.0)
Disease control rate (%) (95% CI)	73.9 (66.7–81.1)
Laboratory data (median)	
CRP (mg/dl)	1.23
Albumin (g/dl)	3.5
Neutrophil (cells/μl)	5395
Lymphocyte (cells/μl)	1285

Abbreviations: ALK, anaplastic lymphoma kinase; BMI, body mass index; CI, confidence interval; CRP, C‐reactive protein; EGFR, epidermal growth factor receptor; PD‐L1, programmed death‐ligand 1; PS, performance status; TPS, tumor proportion score; WT, wild type.

### 3.2 Comparison of the GPS, NLR, and BMI

Table 2 presents the patient characteristics according to the GPS, NLR, and BMI. The GPS values at the initiation of pembrolizumab monotherapy were 0–1 (85 patients) and 2 (57 patients). The ECOG‐PS, clinical stage at diagnosis, liver metastases, bone metastases, and response rate showed statistically significant differences (*p* < 0.05) with the GPS values. The NLR values at the initiation of pembrolizumab monotherapy were low (86 patients) and high (56 patients). The ECOG‐PS, liver metastases, bone metastases, prior radiotherapy, and disease control rate showed statistically significant differences (*p* < 0.05) with the NLR values. The BMI at the initiation of pembrolizumab monotherapy was low (90 patients) and high (52 patients). The administration cycles of pembrolizumab, response rate, and number of lymphocytes exhibited statistically significantly differences (*p* < 0.05) with the BMI.

**TABLE 2:** Results of the patient's characteristics according to GPS, NLR, and BMI

Variables	GPS			NLR			BMI		
	0–1	2	*p*‐value	Low (<5)	High (≥5)	*p*‐value	Low (<21.4)	High (≥21.4)	*p*‐value
Patients (*n*)	85	57		86	56		90	52	
Characteristics									
Gender									
Male/female	68/17	49/8	0.50	70/16	47/9	0.82	74/16	43/9	0.99
Median age at treatment (years) [range]	70 (48–85)	70 (47–86)	0.71^a^	70 (48–86)	69 (47–86)	0.28^a^	70.5 (47–86)	69 (48–84)	0.29^a^
PS									
0–1/≥2	78/7	32/25	**<0.0001**	78/8	32/24	**<0.0001**	67/23	43/9	0.3
Smoking history									
Yes/No	76/9	54/3	0.36	80/6	50/6	0.54	85/5	45/7	0.12
Histology									
Adenocarcinoma/non‐adenocarcinoma	48/37	27/30	0.3	49/37	26/30	0.23	46/44	29/23	0.6
Clinical stage at diagnosis									
III–IV/postoperative recurrence	69/16	54/3	**0.02**	75/11	48/8	0.8	77/13	46/6	0.79
PD‐L1 TPS (%)									
50–89/90–100	50/35	35/22	0.86	55/31	30/26	0.22	56/34	29/23	0.48
Driver mutation/translocation									
EGFR/ALK/WT, negative, unknown	0/0/85	0/0/57	‐	0/0/86	0/0/56	‐	0/0/90	0/0/52	‐
Intracranial metastases at initial treatment									
Yes/No	21/64	13/44	0.84	20/66	14/42	0.84	24/66	10/42	0.41
Liver metastases at initial treatment									
Yes/No	3/82	8/49	**0.02**	3/83	8/48	**0.02**	9/81	2/50	0.32
Bone metastases at initial treatment									
Yes/No	17/68	27/30	**0.0008**	20/66	24/32	**0.01**	27/63	17/35	0.85
BMI (kg/m^2^)									
Median (range)	20.4 (14.1–28.4)	20.0 (16.0–31.7)	0.46^a^	20.6 (14.1–28.4)	19.7 (15.9–31.7)	0.07^a^	‐	‐	‐
Prior radiotherapy									
Yes/No	28/57	17/40	0.71	21/65	24/32	**0.02**	28/62	17/35	0.85
Administration cycles of pembrolizumab									
Median (range)	8 (1–55)	2 (1–31)	0.46^a^	7.5 (1–55)	3 (1–36)	0.21^a^	4 (1–34)	8 (1–55)	**0.0082** ^a^
Tumor response									
Complete response	1	0		1	0		0	1	
Partial response	43	17		40	20		29	31	
Stable disease	24	20		29	15		36	8	
Progressive disease	11	14		13	12		18	7	
Not evaluated	6	6		3	9		7	5	
Response rate (%) (95% CI)	51.7 (41.1–62.3)	29.8 (17.9–41.7)	**0.01**	47.6 (37.1–58.2)	35.7 (23.1–48.2)	0.17	32.2 (22.5–41.8)	61.5 (48.3–74.7)	**0.0008**
Disease control rate (%) (95% CI)	80.0 (71.4–88.5)	64.9 (52.5–77.3)	0.05	81.3 (73.1–89.6)	62.5 (49.8–75.1)	**0.01**	72.2 (62.9–81.4)	76.9 (65.4–88.3)	0.69
Laboratory data									
CRP (mg/dl)	0.38	5	**<0.0001** ^a^	0.71	2.86	**<0.0001** ^a^	1.2	1.7	0.23^a^
Albumin (g/dl)	3.8	3	**<0.0001** ^a^	3.6	3	**<0.0001** ^a^	3.5	3.6	0.43^a^
Neutrophil (cells/μl)	4840	7187	**<0.0001** ^a^	4808	6879	**<0.0001** ^a^	5330	5469	0.45^a^
Lymphocyte (cells/μl)	1410	1073	**0.01** ^a^	1615	863.5	**<0.0001** ^a^	1238	1395	**0.04** ^a^

*p*‐values in bold are statistically significant (*p* < 0.05).

Abbreviations: ALK, anaplastic lymphoma kinase; BMI, body mass index; CI, confidence interval; CRP, C‐reactive protein; EGFR, epidermal growth factor receptor; GPS, Glasgow prognostic score; NLR, neutrophil‐to‐lymphocyte ratio; PD‐L1, programmed death‐ligand 1; PS, performance status; TPS, tumor proportion score; WT, wild type.

^a^ Welch's *t*‐test.

### 3.3 Survival analysis

**FIGURE 1:**
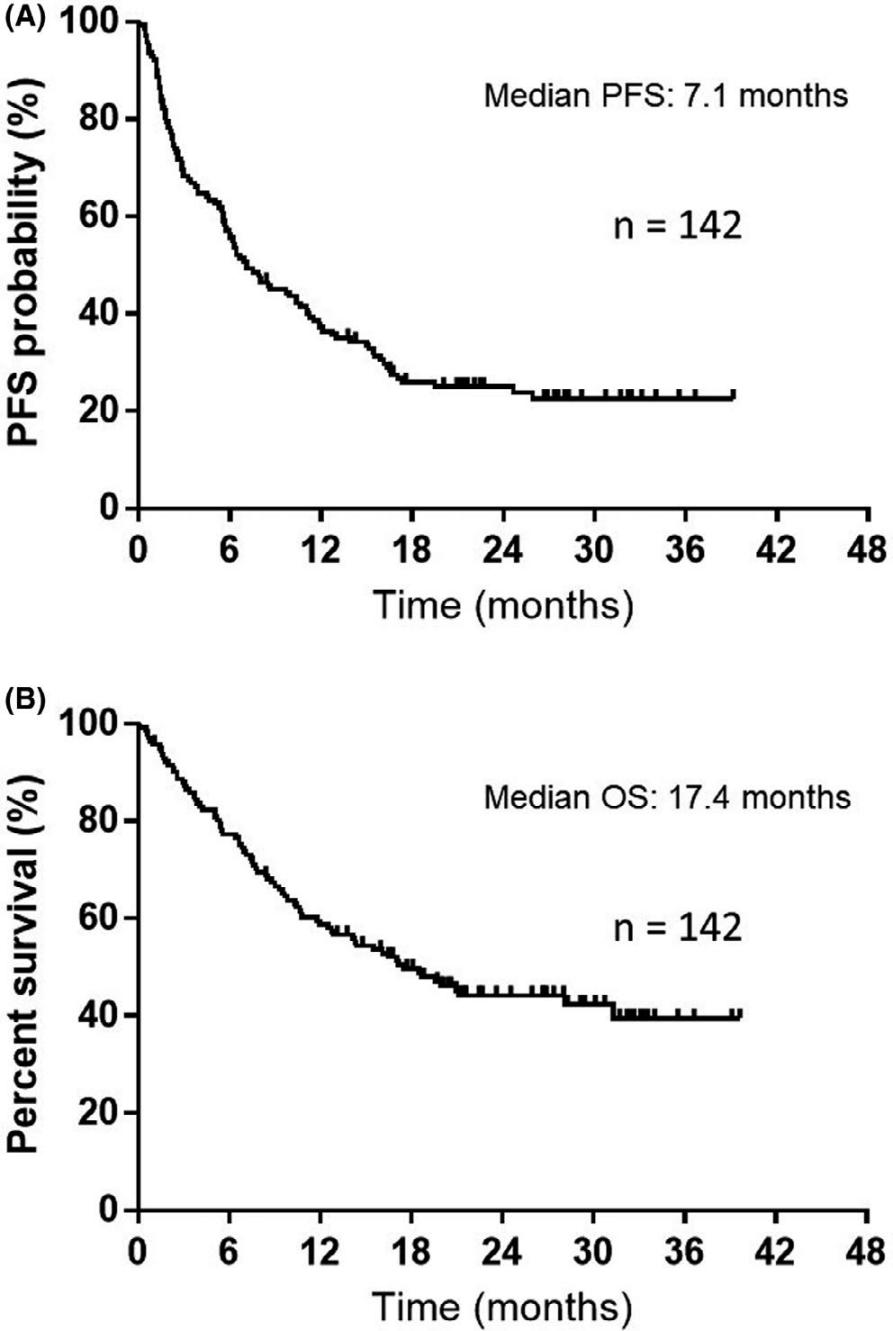
Kaplan–Meier curves for progression‐free survival (PFS) and overall survival (OS). (A) The median PFS was 7.1 months among all 142 patients who received pembrolizumab monotherapy as a first‐line treatment. (B) The median OS was 17.4 months among all 142 patients who received pembrolizumab monotherapy as a first‐line treatment

**FIGURE 2:**
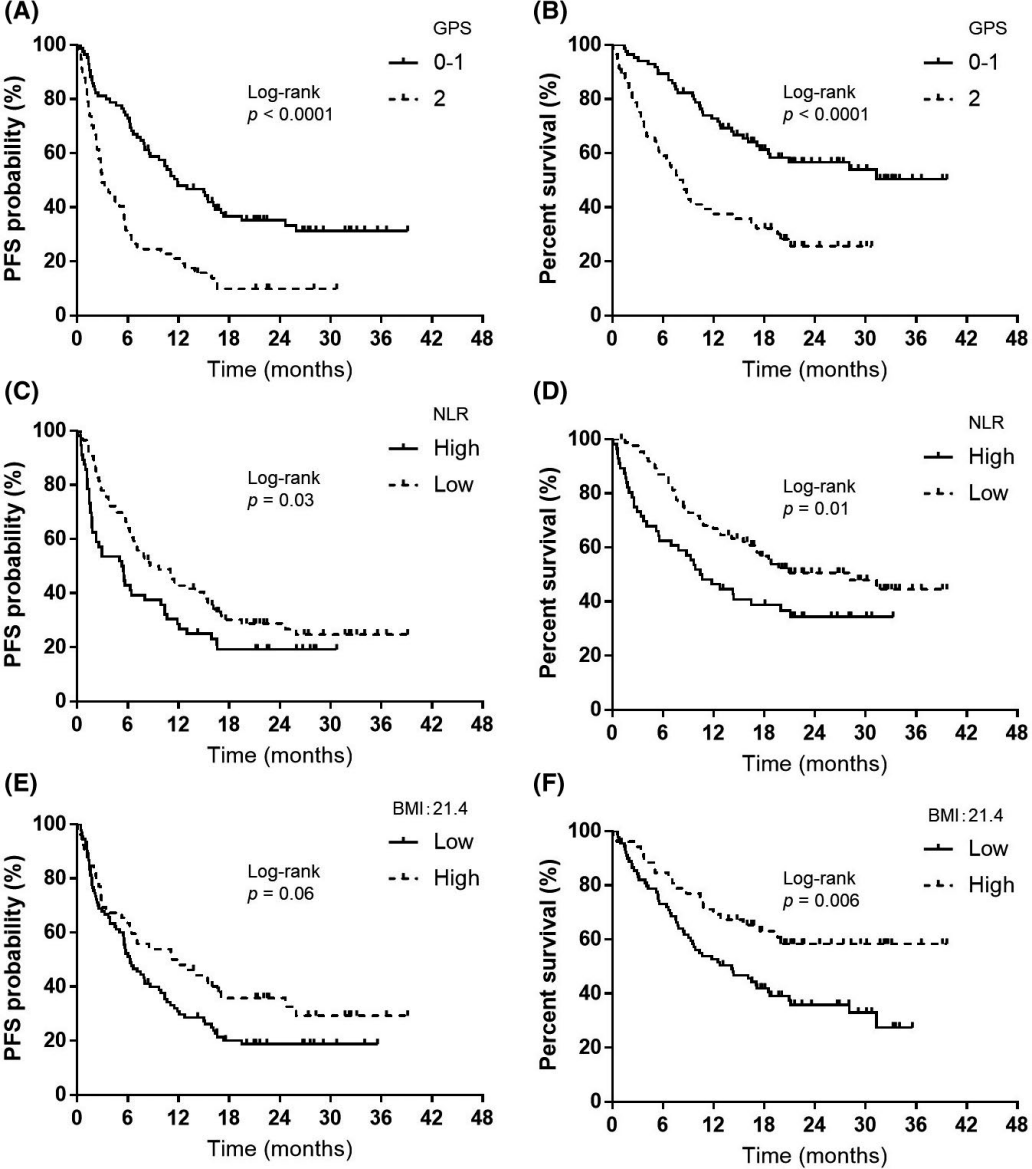
Kaplan–Meier curves for progression‐free survival (PFS) and overall survival (OS) according to Glasgow prognostic score (GPS), neutrophil‐to‐lymphocyte ratio (NLR), and body mass index (BMI). (A) PFS according to GPS at the start of pembrolizumab monotherapy (GPS 0–1, median PFS = 11.8 months; GPS 2, median PFS = 2.9 months). (B) OS according to GPS at the start of pembrolizumab monotherapy (GPS 0–1, median OS = not reached; GPS 2, median OS = 8.3 months). (C) PFS according to NLR at the start of pembrolizumab monotherapy (NLR high, median PFS = 5.3 months; NLR low, median PFS = 8.6 months). (D) OS according to NLR at the start of pembrolizumab monotherapy (NLR high, median OS = 10.5 months; NLR low, median OS = 28.0 months). (E) PFS according to BMI at the start of pembrolizumab monotherapy (BMI high, median PFS = 11.5 months; BMI low, median PFS = 6.2 months). (F) OS according to BMI at the start of pembrolizumab monotherapy (BMI high, median OS = not reached; BMI low, median OS = 14.1 months)

Over a median follow‐up period of 15.7 (range, 0.1–39.6) months, the median PFS interval was 7.1 months (95% CI 5.6–10.6) (Figure 1A) and the median OS interval was 17.4 months (95% CI 12.4–31.3) (Figure 1B). Among the 142 patients, 78 died and 64 were alive at the data cut‐off date of June 30, 2020. Table 3 shows the results of univariate and multivariate analyses of PFS and OS. Univariate analyses of PFS showed significant correlations with the ECOG‐PS, prior radiotherapy, the GPS, and NLR. Multivariate analyses showed that PFS was correlated with prior radiotherapy (HR: 1.57, *p* = 0.03) and a GPS of 0–1 (HR: 0.40, *p* = 0.0002). Furthermore, univariate analyses of OS demonstrated significant correlations with the ECOG‐PS, GPS, NLR, and BMI. Multivariate analyses revealed that OS was associated with a GPS of 0–1/2 (HR: 0.42, *p* = 0.001) and low BMI/high BMI (HR 1.99, *p* = 0.005). Figure 2 presents the Kaplan–Meier curves for PFS and OS, according to the GPS, NLR, and BMI; a GPS of 0–1 was correlated with significantly longer PFS and OS than a GPS of 2 (both, *p* < 0.05; Figure 2A,B). Low NLR was correlated with significantly longer PFS and OS than high NLR (both *p* < 0.05, Figure 2C,D). Although high BMI was not associated with longer PFS than low BMI (*p* = 0.06, Figure 2E), high BMI was associated with significantly longer OS than low BMI (*p* < 0.05, Figure 2F).

**TABLE 3:** Univariate and multivariate analyses of PFS and OS

Variables	Median PFS	Univariate analysis			Multivariate analysis			Median OS	Univariate analysis			Multivariate analysis		
	(months)	HR	95% CI	*p*‐value	HR	95% CI	*p*‐value	(months)	HR	95% CI	*p*‐value	HR	95% CI	*p*‐value
Gender														
Male/female	7.1/6.9	0.86	0.54–1.44	0.57				17.1/20.0	1.09	0.62–2.07	0.76			
Age														
<75/≥75	6.5/7.7	1.21	0.80–1.89	0.36				20.0/10.8	0.82	0.51–1.34	0.42			
PS														
0–1/2–3	9.7/2.9	0.56	0.36–0.88	**0.01**	0.92	0.56–1.56	0.77	20.9/6.7	0.48	0.30–0.81	**0.007**	0.76	0.43–1.37	0.36
Smoking history														
Yes/No	7.0/12.9	1.17	0.62–2.50	0.63				17.5/18.5	1.02	0.50–2.45	0.94			
Histology														
Adenocarcinoma/non‐adenocarcinoma	8.5/6.2	0.87	0.59–1.27	0.47				21.1/14.4	0.72	0.46–1.13	0.15			
Clinical stage at diagnosis														
III–IV/postoperative recurrence	7.1/7.1	1.4	0.78–2.77	0.26				17.5/31.3	1.09	0.59–2.27	0.77			
PD‐L1 TPS (%)														
50–89/90–100	7.1/7.5	1.01	0.68–1.49	0.95				17.1/20.0	1.1	0.70–1.75	0.67			
Intracranial metastases at initial treatment														
Yes/No	8.5/7.1	0.96	0.60–1.48	0.86				20.9/17.1	0.82	0.46–1.40	0.49			
Liver metastases at initial treatment														
Yes/No	2.3/7.9	1.79	0.87–3.28	0.1				9.3/18.7	1.86	0.82–3.64	0.12			
Bone metastases at initial treatment														
Yes/No	5.5/9.7	1.49	0.98–2.20	0.05				14.4/18.7	1.16	0.71–1.850	0.53			
Prior radiotherapy														
Yes/No	5.5/8.5	1.6	1.06–2.36	**0.02**	1.57	1.02–2.36	**0.03**	12.7/21.1	1.46	0.91–2.30	0.11			
GPS														
0, 1/2	11.8/2.9	0.4	0.27–0.59	**<0.0001**	0.4	0.24–0.64	**0.0002**	NR/8.3	0.38	0.24–0.60	**<0.0001**	0.42	0.25–0.71	**0.001**
NLR														
Low (<5)/high (≥5)	8.6/5.3	0.66	0.45–0.97	**0.03**	1.13	0.71–1.83	0.59	28.0/10.5	0.57	0.36–0.89	**0.01**	0.9	0.54–1.50	0.69
BMI (kg/m^2^)														
Low (<21.4)/high (≥21.4)	6.2/11.5	1.45	0.97–2.21	0.06				14.1/NR	1.97	1.21–3.33	**0.005**	1.99	1.21–3.38	**0.005**

The reference arms are the variables shown in the right‐sided arms. *p*‐values in bold are statistically significant (*p* < 0.05).

Abbreviations: BMI, body mass index; CI, confidence interval; GPS, Glasgow prognostic score; HR, hazard ratio; NLR, neutrophil‐to‐lymphocyte ratio; OS, overall survival; PD‐L1, programmed death‐1; PFS, progression‐free survival; PS, performance status; TPS, tumor proportion score.

To further explore factors affecting PFS and OS between patients with a GPS of 0–1 and those with a GPS of 2, we performed a subgroup analysis of the ECOG‐PS by the groups 0–1 and 2–3; histology by adenocarcinoma and non‐adenocarcinoma; PD‐L1 expression by the groups with 50%–89% and 90%–100% expression; NLR by the high‐ (≥5) and low‐ (<5) value groups, BMI by the high‐ (≥21.4) and low‐ (<21.4) BMI groups, and tumor response by PR (CR + PR) and non‐PR (SD + PD) (Table S1). The subgroup analysis showed statistically significant differences in both PFS and OS between a GPS of 0–1 and a GPS of 2 in all groups, except in the ECOG‐PS 2–3 cohort, high NLR cohort, low BMI cohort, and tumor response PR cohort.

## 4 DISCUSSION

The current study evaluated the relationship of the GPS, NLR, and BMI with treatment efficacy among patients with high PD‐L1 expression undergoing first‐line pembrolizumab monotherapy for NSCLC. Multivariate analyses revealed that the GPS and BMI were independently associated with OS, suggesting that the GPS and BMI may be used to predict the OS among patients with high PD‐L1 expression undergoing first‐line pembrolizumab monotherapy for NSCLC. To the best of our knowledge, this is the first study to assess the relationship among the GPS, NLR, and BMI and survival among patients with high PD‐L1 expression undergoing first‐line pembrolizumab monotherapy for NSCLC.

Although ICIs are key drugs for patients with NSCLC with high PD‐L1 expression, a subset of patients does not respond to ICIs. In the present study, the group with a GPS of 0–1 presented a significantly higher response rate and disease control rate than the group with a GPS of 2. In addition, the GPS was significantly predictive of both PFS and OS. The GPS has prognostic importance in lung cancer independent of disease stage and conventionally used prognostic markers^5^, ^6^, ^7^, ^8^, ^9^, ^10^, ^11^, ^12^, ^13^, ^14^; additionally, it has been reported to correlate with elevated cytokine levels, adipokine levels, drug metabolism, weight and muscle loss, and poor PS.^4^, ^29^, ^30^, ^31^, ^32^, ^33^, ^34^, ^35^ These factors may be related to the immune status of the host, and they may affect the efficacy of anti‐programmed cell death protein 1 (PD‐1) therapy. In our analysis, the relationship between patient background and the GPS was significantly related to the ECOG‐PS (0–1/≥2), clinical stage (III–IV/postoperative recurrence), and the presence of metastases such as liver and bone metastases, suggesting that the GPS is affected by these clinical factors. Table 4 summarizes the studies till date that have evaluated the GPS in patients administered anticancer drug therapy for advanced NSCLC. All reports on studies using cytotoxic anticancer drugs, molecularly targeted drugs, and ICIs have indicated the usefulness of the GPS.^5^, ^6^, ^13^, ^14^, ^36^, ^37^, ^38^, ^39^, ^40^ However, although certain reports have incorporated first‐line pembrolizumab monotherapy, no reports have focused on first‐line pembrolizumab monotherapy in patients with high PD‐L1 expression. Furthermore, the GPS is calculated from serum CRP and albumin levels, which indicates that these tests are easily used in clinical practice in most institutions. Multivariate analysis revealed that the GPS, but not the ECOG‐PS, was independently correlated with PFS and OS (Table 3). There are opinions in favor of the GPS being superior to the ECOG‐PS in predicting the prognosis of patients with NSCLC and high PD‐L1 expression who receive first‐line pembrolizumab monotherapy; however, the GPS and ECOG‐PS alone are significantly associated. The ECOG‐PS is a subjective index scoring system that is used to assess the general well‐being of patients with cancer. Conversely, the GPS is an objective and highly reproducible parameter that can be used to classify patients more precisely according to a three‐index‐grading system. Thus, the GPS may be more appropriate for clinical pretreatment assessments.^15^ Most clinical oncologists do not determine the introduction of pembrolizumab monotherapy only on the basis of serum albumin and CRP levels, but they are hesitant to start it for patients with poorer PS. Thus, these two markers have different dimensions and should complement each other. Besides, GPS consisting of a combination of albumin and CRP should be used in a complementary manner by combining the two factors rather than by using them alone. In addition, the assessment of the GPS is more objective than the conventional prognostic factor of the ECOG‐PS.^41^ In this study, we analyzed various patient subgroups according to the ECOG‐PS, histology, PD‐L1 expression, NLR, BMI, and tumor response. We found significant prognostic differences among patients with a GPS of 0–1 and those with a GPS of 2 in most patient subgroups. Therefore, it is reasonable to consider the use of the GPS in clinical practice.

**TABLE 4:** Reports of the Glasgow prognostic score on anticancer drug therapy for advanced non‐small cell lung cancer

Report	Year	Region	Ethnicity	Study type	Sample size	Stage	Treatment	Use of ICIs	Treatment line	Outcomes	Significance of GPS	HR (95% CI)
Forrest et al.^5^	2004	UK	European	Prospective	109	III–IV	Chemotherapy (platinum‐based)	No	Untreated	OS	Yes	GPS 2/0–1: OS; 1.88 (95% CI 1.25–2.84)
Gioulbasanis et al.^6^	2012	Greece	European	Retrospective	96	IV	Chemotherapy (platinum‐based)	No	Untreated	PFS and OS	Yes	GPS 2/0: PFS; 3.78 (95% CI 1.78–8.03), OS; 2.63 (95% CI 1.29–5.34)
Rinehart et al.^36^	2013	USA	Caucasian and African	Prospective	51	IV	Chemotherapy (carboplatin plus gemcitabine)	No	Untreated	OS	Yes	NR
Jiang and Lu^37^	2014	China	Chinese	Prospective	138	III–IV	Chemotherapy (cisplatin‐based)	No	Untreated	PFS and OS	Yes	GPS 2/0: PFS; 0.5 (95% CI 0.3–0.8), OS; 0.5 (95% CI 0.2–0.8)
Fan et al.^38^	2016	China	Chinese	Retrospective	1745	I–IV	Radiotherapy and/or Chemotherapy (any cytotoxic drugs)	No	Untreated	OS	Yes	GPS 2/0–1: OS; 1.872 (95% CI 1.504–2.330)
Kasahara et al.^13^	2019	Japan	Japanese	Retrospective	47	I–IV	Chemotherapy (pembrolizumab, or nivolumab monotherapy)	Yes (ICIs only)	Untreated and treated	PFS and OS	Yes (post treatment GPS)	GPS 0–1/2: PFS; 0.45 (95% CI 0.21–0.99), OS; 0.18 (95% CI 0.06–0.48)
Kasahara et al.^40^	2020	Japan	Japanese	Retrospective	214	I–IV	Chemotherapy (gefitinib, erlotinib, or afatinib)	No (EGFR‐TKIs only)	Untreated and treated	PFS and OS	Yes	GPS 2/0–1: PFS; 1.66 (95% CI 1.03–2.61), OS; 1.77 (95% CI 1.03–2.97)
Pan et al.^39^	2021	China	Chinese	Retrospective	494	III–IV	Chemotherapy (details unknown)	NR	Untreated and treated	OS	Yes	GPS 2/0: OS; 2.09 (95% CI 1.36–3.22)
Takamori et al.^14^	2021	Japan	Japanese	Retrospective	304	III–IV, recurrence	Chemotherapy (pembrolizumab, nivolumab, or atezolizumab monotherapy)	Yes (ICIs only)	Untreated and treated	PFS and OS	Yes	GPS 2/1/0: PFS; 1.37 (95% CI 1.14–1.65), OS; 1.44 (95% CI 1.09–1.90)
Current study		Japan	Japanese	Retrospective	142	III–IV, recurrence	Chemotherapy (pembrolizumab monotherapy)	Yes (ICIs only)	Untreated	PFS and OS	Yes	GPS 0–1/2: PFS; 0.36 (95% CI 0.24–0.64), OS; 0.19 (95% CI 0.25–0.71)

Abbreviations: CI, confidence interval; EGFR‐TKIs, epidermal growth factor receptor‐tyrosine kinase inhibitors; GPS, Glasgow prognostic score; HR, hazard ratio; ICIs, immune checkpoint inhibitors; NR, not reported; OS, overall survival; PFS, progression‐free survival; UK, United Kingdom; USA, United States of America.

Furthermore, the GPS is associated with survival in patients receiving not only ICIs, but also in those receiving cytotoxic agents. Thus, GPS has an aspect of prognostic factor similar to PS. If GPS is solely a prognostic factor and does not affect the survival as a predictive factor, it may not contribute to the selection of treatment options. For example, if a prognosis would be poor in patients with poor GPS for any treatment, such as ICI monotherapy, combination therapy with ICIs plus cytotoxic agents, or cytotoxic agents, GPS itself may not be useful for the selection of treatment options. In the present study, we cannot draw a conclusion whether GPS is a predictive or prognostic factor because we did not include patients who received other treatments, including cytotoxic agent or combination therapy with ICIs and cytotoxic agents. However, we cannot exclude the possibility that GPS might be a predictive factor for survival of patients receiving pembrolizumab monotherapy. Furthermore, even if GPS is a prognostic factor rather than a predictive factor, it can contribute to the selection of treatment in clinical practice settings.

Several studies have demonstrated the relationship of NLR with clinical response and outcomes in patients with NSCLC treated with anti‐PD‐1 inhibitors.^42^, ^43^ For example, NLR may be able to predict the prognosis of patients with NSCLC treated with nivolumab.^20^ In our analysis, the relationship between patient background and NLR was significantly related to the ECOG‐PS (0–1/≥2), the presence of metastases such as liver and bone metastases, and prior radiotherapy, suggesting that NLR is affected by these clinical factors. Although there was no significant difference in the response rate between the low‐NLR and high‐NLR groups, the disease control rate was significantly higher in the low‐NLR group. Furthermore, although log‐rank tests showed that low NLR was associated with significantly longer PFS and OS than high NLR, according to the multivariate analysis, the NLR did not correlate with either PFS or OS in patients with high PD‐L1 expression treated with first‐line pembrolizumab monotherapy. These results indicate that NLR did not significantly affect PFS and OS in our patient cohort.

Regarding BMI, a large cohort retrospective study demonstrated that a high BMI is correlated with longer PFS and OS beyond ICI administration in patients with metastatic melanoma.^44^ Another retrospective study demonstrated that BMI is correlated with ICI efficacy in solid malignant tumors, including melanoma, renal cell carcinoma, and NSCLC.^24^ In addition, a study has shown a relationship between BMI and ICI outcomes in patients with NSCLC.^25^ The study demonstrated that BMI was significantly associated with the efficacy of ICIs in patients with NSCLC treated with second‐ or later‐line PD‐1/PD‐L1 inhibitors. However, according to that report, PFS and OS were not significantly different between high‐ and low‐BMI groups of patients with NSCLC and high PD‐L1 expression (≥50%) who were treated with pembrolizumab as first‐line therapy. The reason for this result may be that their study consisted of 84 patients with high PD‐L1 expression (≥50%), which may have been an insufficient number for detecting a statistically significant difference. In the current analysis, the patient background was not significantly different between the high‐ and low‐BMI groups, except for administration cycles of pembrolizumab and lymphocyte count. Although there was no significant difference in the disease control rate between the low‐BMI and high‐BMI groups, the response rate was significantly higher in the high‐BMI group. Furthermore, the BMI was significantly predictive of OS but not of PFS. This may indicate that a higher BMI not only increases the efficacy of pembrolizumab monotherapy in these patients, but it may also provide an opportunity for patients to receive additional treatment cycles of pembrolizumab.

The current study has several limitations. First, the retrospective study design relied on subjective physician evaluations of treatment response, which may have introduced variability in the data regarding response and PFS. Second, the sample size was relatively small; however, this would be an inherent limitation at most centers that generally do not have many patients with high PD‐L1 expression who are undergoing first‐line pembrolizumab monotherapy for NSCLC. Thus, it is important to consider the potential significance of these sources of bias when interpreting our data. Third, the cut‐off values for laboratory data or BMI have not been established, as there were various cut‐off values in previous studies. In our analysis, for the GPS and NLR, we used the cut‐off values reported previously; for BMI, we determined the cut‐off values using ROC curves. Therefore, it is necessary to examine whether these values are clinically valid for a larger population in the future.

In conclusion, the results of this investigation suggest that the GPS is independently associated with PFS and OS. In addition, BMI was independently associated with OS. Therefore, our results should be evaluated in larger studies to determine whether they are generalizable to other patient populations. Although further studies are warranted to validate these findings, our results suggest that determination of the GPS and BMI may aid in predicting treatment outcome for patients with NSCLC and high PD‐L1 expression who are administered first‐line treatment with pembrolizumab monotherapy.

## CONFLICT OF INTEREST

Kyoichi Kaira has received research grants and a speaker honorarium from Ono Pharmaceutical Company, Boehringer Ingelheim, Chugai Pharmaceutical, Taiho Pharmaceutical, Eli Lilly Japan, and AstraZeneca. Atsuto Mouri has received a speaker honorarium from Eli Lilly, Taiho Pharmaceutical, Pfizer, Chugai Pharmaceutical, and AstraZeneca. Hiroshi Kagamu has received research grants and a speaker honorarium from Ono Pharmaceutical Company, Bristol‐Myers Company, Boehringer Ingelheim, MSD, Daiichi Sankyo Company, Chugai Pharmaceutical, Taiho Pharmaceutical, Merck Biopharma Company, Eli Lilly Japan, and AstraZeneca. Kunihiko Kobayashi has received research grants and a speaker honorarium from Boehringer Ingelheim, AstraZeneca, and Bristol‐Myers Company.

## ETHICAL APPROVAL STATEMENT

All procedures complied with the ethical standards of the institutional and/or national research committee and with the 1964 Helsinki Declaration and its later amendments or comparable ethical standards. The study was approved by the ethics committee of Saitama Medical University International Medical Center (No. 20–222). The requirement for informed consent was waived owing to the retrospective nature of the study.

## CLINICAL TRIAL REGISTRATION NUMBER

Not applicable.

## Supporting information

Table S1

## Data Availability

The data that support the findings of this study are available on request from the corresponding author. The data are not publicly available due to privacy or ethical restrictions.
