# Supplementary material for: Pretreatment Glasgow prognostic score predicts survival among patients with high PD‐L1 expression administered first‐line pembrolizumab monotherapy for non‐small cell lung cancer
Source: Cancer Med. 2021 Aug 20;10(20):6971–84. doi: 10.1002/cam4.4220 (PMC8525165; doi:10.1002/cam4.4220)
Supplement: Supplementary file 1 — Table S1 [file CAM4-10-6971-s001.doc]

Supplementary Table 1. Comparison of progression-free survival (PFS) and overall survival (OS) for Glasgow prognostic score (GPS) in various subgroups

| Variables | Median PFS (months) | Univariate analysis | | | Median OS (months) | Univariate analysis | | |
| --- | --- | --- | --- | --- | --- | --- | --- | --- |
|  | GPS: 0–1/2 | HR | 95% CI | *p*-value | GPS: 0–1/2 | HR | 95% CI | *p*-value |
| Performance status (PS) |  |  |  |  |  |  |  |  |
| 0–1 | 11.9/3.5 | 0.35 | 0.22–0.56 | **0.0001** | NR/9.1 | 0.38 | 0.22–0.66 | **0.0008** |
| 2–3 | 6.7/2.8 | 0.82 | 0.30–1.93 | 0.67 | 14.4/5.6 | 0.63 | 0.18–1.70 | 0.38 |
| Histology |  |  |  |  |  |  |  |  |
| Adenocarcinoma | 11.5/2.9 | 0.44 | 0.26–0.77 | **0.004** | NR/10.8 | 0.41 | 0.21–0.79 | **0.009** |
| Non-adenocarcinoma | 11.9/3.3 | 0.36 | 0.20–0.64 | **0.0005** | 28.1/6.0 | 0.37 | 0.196–0.69 | **0.001** |
| PD-L1 TPS (%) |  |  |  |  |  |  |  |  |
| 50–89 | 11.5/3.0 | 0.39 | 0.24–0.65 | **0.0004** | 31.3/8.5 | 0.36 | 0.20–0.65 | **0.0007** |
| 90–100 | 11.9/3.1 | 0.42 | 0.22–0.79 | **0.0078** | NR/8.1 | 0.42 | 0.20–0.87 | **0.02** |
| NLR |  |  |  |  |  |  |  |  |
| High (≥5) | 7.1/2.9 | 0.77 | 0.41–1.40 | 0.41 | 14.4/6.3 | 0.49 | 0.23–0.99 | **0.04** |
| Low (<5) | 15.5/3.3 | 0.19 | 0.10–0.34 | **0.0001** | NR/8.7 | 0.35 | 0.19–0.68 | **0.002** |
| BMI (kg/m^2^) |  |  |  |  |  |  |  |  |
| High (≥21.4) | 9.7/3.8 | 0.48 | 0.30–0.78 | **0.003** | 20.9/6.8 | 0.35 | 0.20–0.61 | **0.0002** |
| Low (<21.4) | 24.7/2.8 | 0.28 | 0.14–0.571 | **0.0006** | NR/19.6 | 0.42 | 0.17–1.01 | 0.05 |
| Tumor response |  |  |  |  |  |  |  |  |
| Partial response | 25.9/12.8 | 0.48 | 0.23–1.02 | 0.05 | NR/NR | 0.64 | 0.26–1.72 | 0.36 |
| Non-partial response | 6.2/2.3 | 0.43 | 0.26–0.70 | **0.0007** | 16.1/5.2 | 0.38 | 0.22–0.65 | **0.0004** |

PFS, progression-free survival; OS, overall survival; GPS, Glasgow prognostic score; HR, hazard ratio; CI, confidence interval; PS, performance status; PD-L1, programmed death-ligand 1; TPS, tumor proportion score; NLR, neutrophil-to-lymphocyte ratio; BMI, body mass index

Partial response includes complete response and partial response, Non-partial response includes stable disease and progressive disease.

The reference arms are the variables shown in the right-sided arms.

*p-*values in bold are statistically significant (*p* < 0.05)
